# Photoluminescent Layered Crystal Consisting of Anderson-Type Polyoxometalate and Surfactant toward a Potential Inorganic–Organic Hybrid Laser

**DOI:** 10.3390/ijms25010345

**Published:** 2023-12-26

**Authors:** Ayaka Mihara, Tatsuhiro Kojima, Yoriko Suda, Kyoka Maezawa, Toshiyuki Sumi, Naoyuki Mizoe, Ami Watanabe, Hironori Iwamatsu, Yoshiki Oda, Yosuke Okamura, Takeru Ito

**Affiliations:** 1 Department of Chemistry, School of Science, Tokai University, Hiratsuka 259-1292, Japan; 2 Department of Applied Chemistry, Kobe City College of Technology, Kobe 651-2194, Japan; 3 Department of Electric and Electronic Engineering, School of Engineering, Tokyo University of Technology, Hachioji 192-0982, Japan; 4 Department of Applied Chemistry, School of Engineering, Tokai University, Hiratsuka 259-1292, Japan; 5 Technology Joint Management Office, Tokai University, Hiratsuka 259-1292, Japan

**Keywords:** inorganic–organic, single crystal, polyoxometalate, surfactant, photoluminescence

## Abstract

The hybridization of inorganic and organic components is a promising strategy to build functional materials. Among several functions, luminescence is an important function which should be considered for practical usage. Inorganic–organic hybrid luminescent materials have been investigated as phosphors, sensors, and lasers. Organic luminescent centers such as dye molecules have often been hybridized with inorganic matrices. Polyoxometalate anions (POMs) are effective inorganic luminescent centers due to their luminescent properties and structural designability. However, most luminescent POM components are limited to lanthanide-based POMs. In this report, a photoluminescent inorganic–organic hybrid crystal based on a non-lanthanide POM was successfully synthesized as a single crystal. Anderson-type hexamolybdochromate ([CrMo_6_O_18_(OH)_6_]^3−^, CrMo_6_) anion exhibiting emission derived from Cr^3+^ was utilized with *n*-dodecylammonium ([C_12_H_25_NH_3_]^+^, C_12_NH_3_) surfactant cation to obtain a photoluminescent hybrid crystal. The grown single crystal of C_12_NH_3_-CrMo_6_ comprised a distinct layered structure consisting of inorganic CrMo_6_ layers and interdigitated C_12_NH_3_ layers. In the CrMo_6_ layers, the CrMo_6_ anions were associated with water molecules by hydrogen bonding to form a densely packed two-dimensional network. Steady-state and time-resolved photoluminescence spectroscopy revealed that the C_12_NH_3_-CrMo_6_ hybrid crystal exhibited characteristic emission from the CrMo_6_ anion. Preliminary lasing properties were also observed for C_12_NH_3_-CrMo_6_, which shows the possibility of using the C_12_NH_3_-CrMo_6_ hybrid crystal as an inorganic–organic hybrid laser.

## 1. Introduction

Synthetic methodology is crucial for the construction of functional compounds and/or materials. The hybridization of inorganic and organic components is a promising strategy to build functional materials [1,2,3]. Unprecedented functions can emerge from the combination of inorganic and organic components. Inorganic motifs can contribute to thermal stability and elemental variation, whereas organic motifs enable flexible molecular design. The synergy of inorganic and organic characteristics has been realized in conductive [4], adsorbent [5], and magnetic materials [6].

Luminescence is another important function which should be considered for several reasons. Inorganic–organic hybrid materials have been investigated as phosphors, sensors, and lasers [7,8,9,10]. Organic luminophores or dyes are often utilized in inorganic matrices [11]. Several inorganic–organic hybrids contain inorganic luminescent centers such as lanthanides, which behave as distinct luminescent centers exhibiting sharp spectra due to the transitions of inner-shell 4f electrons [12,13,14,15].

**Figure 1:**
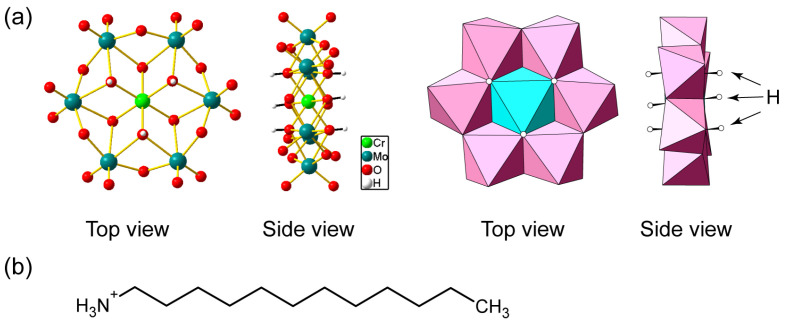
(**a**) Molecular structure of hexamolybdochromate (CrMo_6_) anion. Mo: teal, Cr: light green, O: red, H: white in ball-and-stick representation, Mo: pink, and Cr: light blue in polyhedral representation; (**b**) molecular structure of *n*-dodecylammonium (C_12_NH_3_) cation.

Polyoxometalate molecular clusters (POMs) are also effective inorganic luminescent centers due to their luminescent properties and structural designability [16,17,18]. Lanthanide-containing POMs [19,20,21] are typically employed, and several luminescent inorganic–organic hybrids have been reported [22,23]. Anderson-type POMs [24,25,26] are another class of luminescent POMs. Hexamolybdochromate [CrMo_6_O_18_(OH)_6_]^3−^ (CrMo_6_, Figure 1a) anion comprises Cr^3+^ surrounded by six oxygen atoms exhibiting red emission similar to ruby lasers [27,28,29,30,31]. In addition, Anderson-type POMs possess planar molecular shapes leading to layered structures [32,33,34,35]. Layered packing of luminescent species gives rise to anisotropic emission, which is beneficial for lasing properties. However, Anderson-type POMs have rarely been employed as luminescent centers in inorganic–organic hybrid materials [30,31,35].

In this report, we constructed an inorganic–organic hybrid with a luminescent Anderson-type CrMo_6_ anion and surfactant cation. The use of *n*-dodecylammonium ([C_12_H_25_NH_3_]^+^, C_12_NH_3_, Figure 1b) derived from aliphatic primary amines enabled the crystallization of the CrMo_6_-surfactant hybrid as single crystals. The C_12_NH_3_-CrMo_6_ hybrid crystal possessed a distinct layered structure due to the planar CrMo_6_ anion and the structure-directing C_12_NH_3_ surfactant. The emission properties, including the preliminary lasing behavior, were investigated.

## 2. Results

### 2.1. Synthesis of C_12_NH_3_-CrMo_6_ Hybrid Crystal

**Figure 2:**
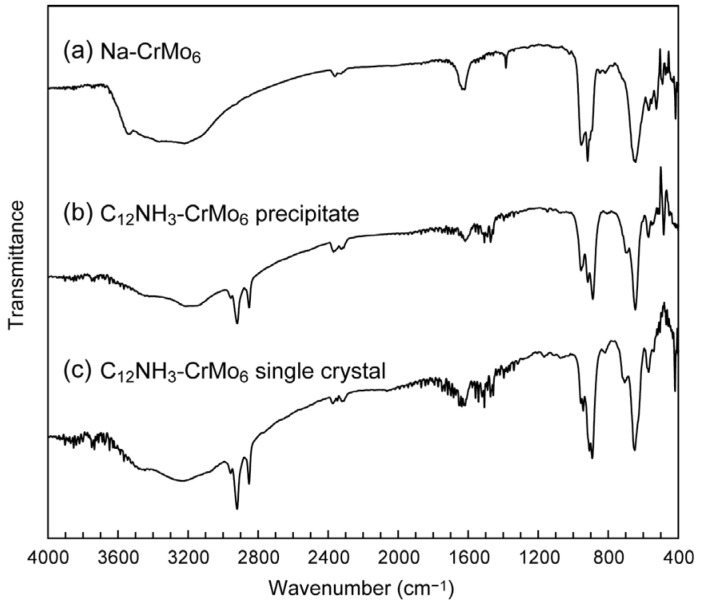
IR spectra of C_12_NH_3_-CrMo_6_ hybrid crystal: (**a**) starting material of Na-CrMo_6_; (**b**) precipitate of C_12_NH_3_-CrMo_6_; (**c**) single crystal of C_12_NH_3_-CrMo_6_.

The C_12_NH_3_-CrMo_6_ hybrid crystal was obtained by an ion exchange reaction between the sodium salt of CrMo_6_ (Na_3_[CrMo_6_O_18_(OH)_6_]·8H_2_O, Na-CrMo_6_) and C_12_NH_3_ cations. The single crystals of C_12_NH_3_-CrMo_6_ were successfully grown from the synthetic filtrate after the removal of a precipitate of the C_12_NH_3_-CrMo_6_ hybrid crystal. The IR spectra (Figure 2) exhibited characteristic peaks of CrMo_6_ (400–1100 cm^−1^), which were assigned as terminal Mo–O_t_ bonds (950–890 cm^−1^) and bridging Mo–O–Mo bonds (700–400 cm^−1^) [36,37,38]. Characteristic peaks of C_12_NH_3_ were observed in the range of 2800–3000 cm^−1^, 2920 cm^−1^ for *ν*_as_(−CH_2_−), and 2850 cm^−1^ for *ν*_s_(−CH_2_−), respectively. The almost identical IR spectra of the precipitate (Figure 2b) and single crystal (Figure 2c) indicated that the precipitate and single crystal of the C_12_NH_3_-CrMo_6_ hybrid were the same in terms of their molecular structures.

**Figure 3:**
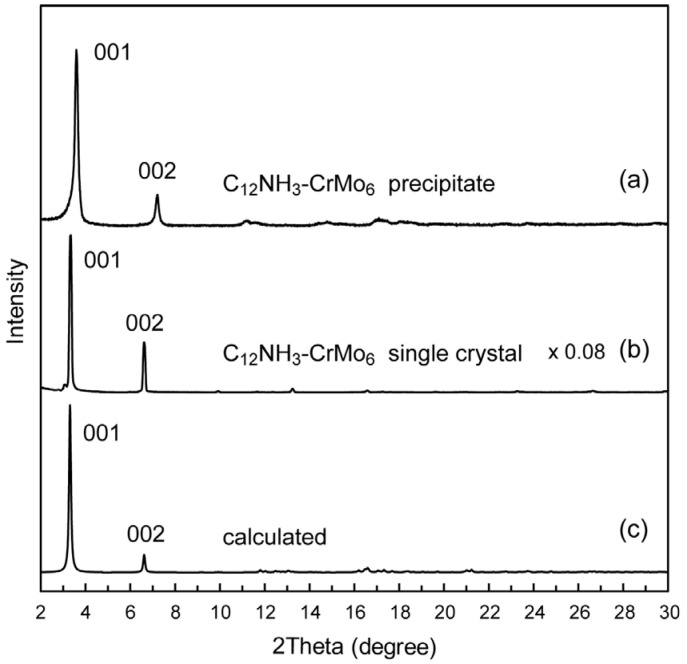
Powder X-ray diffraction patterns of C_12_NH_3_-CrMo_6_ hybrid crystal: (**a**) precipitate of C_12_NH_3_-CrMo_6_; (**b**) single crystal of C_12_NH_3_-CrMo_6_; (**c**) calculated pattern of C_12_NH_3_-CrMo_6_ from the structure revealed by single-crystal X-ray diffraction.

The precipitate and single crystal of the C_12_NH_3_-CrMo_6_ hybrid crystal exhibited slightly different XRD patterns (Figure 3a,b). Both XRD patterns showed strong peaks assignable to 001 and 002 reflections in the lower 2-theta range featuring layered materials. Estimated interlayer spacing was 24.5 Å for the C_12_NH_3_-CrMo_6_ precipitate and 26.6 Å for the C_12_NH_3_-CrMo_6_ single crystal, respectively. The XRD pattern of the C_12_NH_3_-CrMo_6_ single crystal was similar to that calculated from the single-crystal X-ray analysis data (Figure 3c). Subtle differences in the peak intensity and position of the patterns may be due to the difference in the measurement temperature (powder: room temperature, single crystal: 103 K), and to the preferred orientation derived from the predominant layered structure of C_12_NH_3_-CrMo_6_.

### 2.2. Crystal Structure of C_12_NH_3_-CrMo_6_ Hybrid Crystal

**Figure 4:**
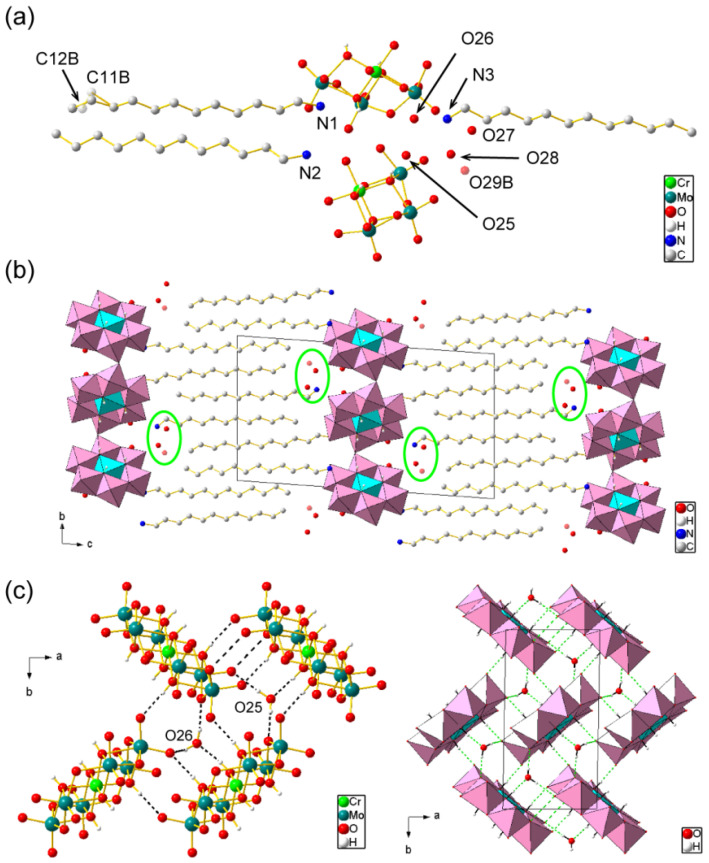
Crystal structure of C_12_NH_3_-CrMo_6_ (Mo: teal, Cr: light green, C: gray, N: blue, O: red, and H: white). H atoms of C_12_NH_3_ cations and solvents were omitted for clarity: (**a**) asymmetric unit. Disordered atoms (C11B, C12B, and O29B) in the minor part are indicated in transparent color; (**b**) packing diagram along *a*-axis. CrMo_6_ anions are depicted in a polyhedral model (Mo: pink; Cr: light blue). Some solvents (O27, O28, and O29B) are highlighted by green circles; (**c**) molecular arrangement of the inorganic monolayer (*ab* plane) in ball-and-stick (left) and polyhedral (right) representations. Broken lines represent short contacts including O–H···O hydrogen bonding between the CrMo_6_ anions and solvents.

The presence and molecular structure of the CrMo_6_ anion were revealed via X-ray structural analysis (Table 1, Figure 4). The asymmetric unit consisted of two half-anions of CrMo_6_ and three C_12_NH_3_ cations including water of crystallization (Figure 4a). This indicates that one CrMo_6_ anion (3− charge) was connected with the three C_12_NH_3_ cations due to charge compensation, which was verified by CHN elemental analysis. Four water molecules (O25, O26, O27, and O28) were assigned unambiguously. Some terminal atoms (C11 and C12) in the C_12_NH_3_ dodecyl chain were disordered, and a small electron density (2.62 eÅ^−3^) remained ca. 2.8 Å away from C12A in the main part of the disordered atoms (Supplementary Material, Figure S1). We assigned this small electron density to a disordered water molecule (O29B) in the minor disordered part together with C11B and C12B, which was refined to have a site occupancy of 0.32. Therefore, the C_12_NH_3_-CrMo_6_ single crystal contained 4.32 water molecules per CrMo_6_ anion, leading to the chemical formula of [C_12_H_25_NH_3_]_3_[CrMo_6_O_18_(OH)_6_]·4.32H_2_O. The six H atoms in the hydroxo group of CrMo_6_ were identified using the X-ray diffraction analysis and bond valence sum (BVS) calculations [39]. The BVS values of the protonated O atoms (O8, O11, O12, O16, O23, and O24) in CrMo_6_ were in the range of 1.19–1.28, whereas those for other O atoms were in the range of 1.65–1.86.

The crystal of C_12_NH_3_-CrMo_6_ was composed of alternating CrMo_6_ inorganic monolayers and C_12_NH_3_ organic bilayers parallel to the *ab* plane (Figure 4b), as typically observed for surfactant-POM hybrid single crystals [40]. The layered periodicity was 26.7 Å, which was consistent with the powder XRD pattern (Figure 3b). The aliphatic chains of C_12_NH_3_ were interdigitated in a straight line. Some solvent water molecules (O27, O28, and O29B) were located at the interface between the CrMo_6_ and C_12_NH_3_ layers (Figure 4b). On the other hand, two water molecules (O25 and O26) were located inside the inorganic CrMo_6_ monolayer. These CrMo_6_ anions and water molecules were densely packed to form a two-dimensional network via short contacts, including O–H⋯O hydrogen bonding (Figure 4c) [41]. The O⋯O distance ranged from 2.71 to 3.00 Å (mean value: 2.80 Å). The hydrophilic heads of C_12_NH_3_ did not penetrate the densely packed CrMo_6_-H_2_O monolayers, but were located at the dip between the CrMo_6_ anions [42] by forming N–H⋯O hydrogen bonds with distances of 2.71–2.84 Å (mean value: 2.78 Å) [41].

### 2.3. Photoluminescent Properties of C_12_NH_3_-CrMo_6_ Hybrid Crystal

**Figure 5:**
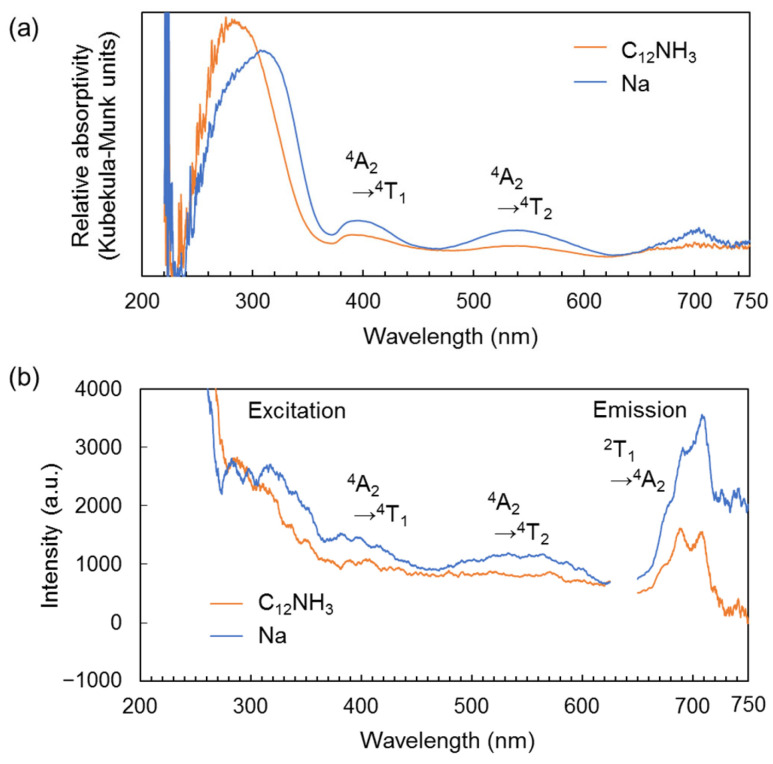
Steady-state spectra of C_12_NH_3_-CrMo_6_ and Na-CrMo_6_ (starting material). The measurement temperature was 300 K: (**a**) diffuse reflectance spectra; (**b**) excitation and emission spectra. Excitation spectra were monitored for the emission at 705 nm. Emission spectra were measured with an excitation wavelength of 300 nm.

As mentioned above, the CrMo_6_ anion exhibits a distinct red emission due to the presence of Cr^3+^ like ruby lasers [27,28,29,30,31]. The photoluminescent properties of C_12_NH_3_-CrMo_6_ were evaluated using steady-state and time-resolved spectroscopy. The diffuse reflectance spectra (Figure 5a) of both C_12_NH_3_-CrMo_6_ and Na-CrMo_6_ (starting material) showed broad peaks around 390 and 540 nm, corresponding to the transitions of the *d*^3^ electron in Cr^3+^ from the ^4^A_2_ ground state to the ^4^T_1_ and ^4^T_2_ excited states, respectively [27]. These electron transitions were also observed in the excitation spectra of C_12_NH_3_-CrMo_6_ and Na-CrMo_6_, as shown in Figure 5b. Characteristic emission derived from Cr^3+^ was observed around 680–710 nm (Figure 5b), which was investigated in detail using time-resolved spectroscopy.

**Figure 6:**
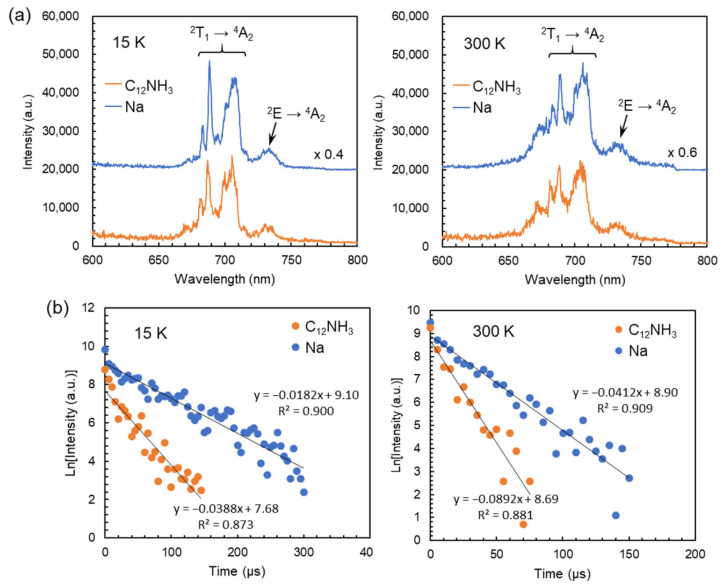
Photoluminescence properties of C_12_NH_3_-CrMo_6_ and Na-CrMo_6_ (starting material) investigated using time-resolved spectroscopy. Each spectrum or decay profile was obtained using a single-pulse excitation with a wavelength of 266 nm: (**a**) emission spectra measured at 15 K (**left**) and 300 K (**right**) with an acquisition time of 0–50 μs; (**b**) emission decay profiles measured at 15 K (**left**) and 300 K (**right**) on the emission at 688 nm.

Figure 6a shows the emission spectra of C_12_NH_3_-CrMo_6_ and Na-CrMo_6_ obtained using a single pulse excitation. Emission peaks at around 682, 688, 701, and 707 nm were derived from the ^2^T_1_ → ^4^A_2_ transition known as “R-lines” [27,28,29,30,31]. The peaks at around 730 nm were assigned to the ^2^E → ^4^A_2_ transition. The spectral shapes of C_12_NH_3_-CrMo_6_ were almost identical to those of Na-CrMo_6_ irrespective of the measurement temperatures. On the other hand, the emission intensity of C_12_NH_3_-CrMo_6_ was lower than that of Na-CrMo_6_ (Figure 6a), and the emission of C_12_NH_3_-CrMo_6_ decayed faster than that of Na-CrMo_6_ at each temperature (Figure 6b). The estimated emission lifetimes were 26 μs (15 K) and 11 μs (300 K) for C_12_NH_3_-CrMo_6_ and 55 μs (15 K) and 24 μs (300 K) for Na-CrMo_6_.

**Figure 7:**
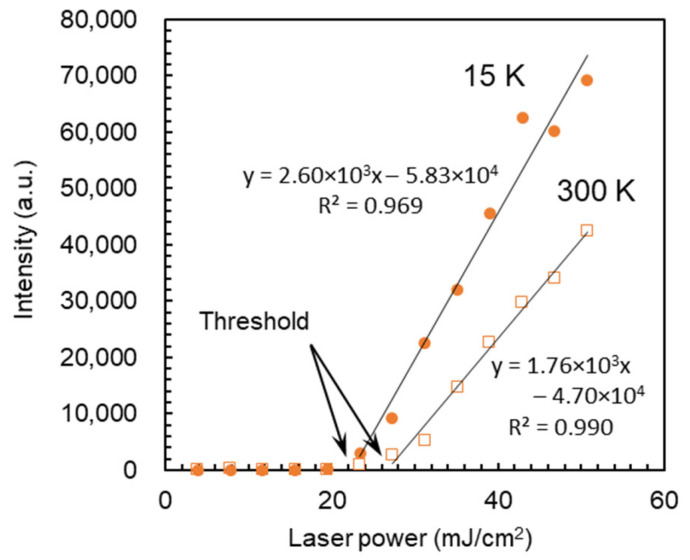
Emission intensity-excitation laser power dependency of C_12_NH_3_-CrMo_6_ at 15 and 300 K. Each data point was obtained using a single-pulse excitation with a wavelength of 266 nm on the emission at 688 nm. Data acquisition time: 0–50 μs.

The title compound C_12_NH_3_-CrMo_6_ had a distinct layered structure together with characteristic emission as described above, which is associated with the plausible emergence of lasing properties. To evaluate the possibility of inorganic–organic hybrid laser materials, the emission intensity of C_12_NH_3_-CrMo_6_ was explored by changing the excitation laser power. Figure 7 shows the emission intensity-excitation laser power dependency at 15 and 300 K. The emission intensity increased linearly with an increase in the excitation laser power above the threshold value, which indicates the emergence of preliminary lasing properties [43,44,45,46]. The threshold values were 22.4 mJ cm^−2^ for 15 K and 26.7 mJ cm^−2^ for 300 K, respectively.

## 3. Discussion

We first synthesized a photoluminescent polyoxometalate-surfactant hybrid crystal by using an Anderson-type CrMo_6_ anion and C_12_NH_3_ surfactant cation. Using primary alkylammonium cations seems essentially effective for the crystallization of the CrMo_6_ anion with surfactant cations. The powder XRD patterns of the C_12_NH_3_-CrMo_6_ hybrid crystal (Figure 3) suggest that the precipitates and single crystals of the C_12_NH_3_-CrMo_6_ hybrid crystal had slightly different phases in their interlayer periodicities. The interlayer spacing was 24.5 Å for the precipitate and 26.6 Å for the single crystal. The precipitate and single crystal possessed distinct layered structures, both of which are believed to be essentially similar, except for the interlayer distances. The difference is derived from the desorption of water molecules (O27, O28, and O29B highlighted in Figure 4b) from the crystalline lattice, shrinking the layered distance to 24.5 Å of the precipitate from 26.6 Å of the single crystal.

Single-crystal X-ray diffraction analysis confirmed a distinct layered structure consisting of the CrMo_6_ inorganic layers and C_12_NH_3_ organic layers. Notably, the CrMo_6_ anions formed a two-dimensional infinite layer with the water molecules through O–H⋯O hydrogen bonding in the CrMo_6_ inorganic layers. The hydrophilic heads of the C_12_NH_3_ surfactant did not penetrate the inorganic CrMo_6_-H_2_O monolayers as observed in another POM crystal hybridized with C_12_NH_3_ [42]. This implies that a densely packed POM inorganic layer is easily formed by using a primary alkylammonium cation with a smaller hydrophobic head [40].

The photoluminescent properties of C_12_NH_3_-CrMo_6_ were investigated by steady-state and time-resolved spectroscopy, revealing the characteristic ruby-like emission derived from Cr^3+^ in the CrMo_6_ anion. As shown in Figure 6a, the emission spectra of C_12_NH_3_-CrMo_6_ were almost identical in shape to those of Na-CrMo_6_ irrespective of the measurement temperature. This suggests that the crystal fields of Cr^3+^ in C_12_NH_3_-CrMo_6_ and Na-CrMo_6_ were similar due to the structural rigidity of the CrMo_6_ anion. The spectral shape of the C_12_NH_3_-CrMo_6_ emission was similar to that of previous compounds containing the CrMo_6_ anion [29,30,31]. On the other hand, the emission lifetimes of C_12_NH_3_-CrMo_6_ (26 μs at 15 K and 11 μs at 300 K) were shorter than those (55 μs at 15 K and 24 μs at 300 K) of Na-CrMo_6_ and another inorganic–organic hybrid consisting of CrMo_6_ anions (240 μs) [30]. These results indicate that the emission efficiency of the CrMo_6_ anion depends on the countercation and that the C_12_NH_3_ cation reduces the emission efficiency. In C_12_NH_3_-CrMo_6_, nonradiative deactivation of the excitation energy (O → Mo ligand-to-metal charge transfer) occurs more easily than in Na-CrMo_6_ through the vibration states of the high-frequency C–H oscillators in C_12_NH_3_ [35].

The distinct layered structure of the C_12_NH_3_-CrMo_6_ hybrid crystal is beneficial for the emergence of lasing properties. Preliminary lasing behavior was observed by changing the excitation laser power, exhibiting the lasing threshold values of 22.4 mJ cm^−2^ at 15 K and 26.7 mJ cm^−2^ at 300 K, respectively (Figure 7). These threshold values were much larger than those of recent organic lasers (order of μJ cm^−2^) [44,45,46], indicating a lower lasing efficiency in C_12_NH_3_-CrMo_6_, probably due to the use of randomly oriented crystalline powder [47]. In the future, the fabrication of large single crystals [48,49] in higher yields or the construction of microcavity structures [47] will be a promising strategy to improve the lasing emission efficiency. The thermal gravimetric (TG) profiles (Figure S2) suggest that the hybrid crystal of C_12_NH_3_-CrMo_6_ started to decompose from ca. 423 K (150 °C) due to the removal of the C_12_NH_3_ cation. A temperature of 423 K (150 °C) may be rather low for the removal of the C_12_NH_3_ cation, and the details of this phenomenon are still unclear. Our previous results showed that three hybrid crystals consisting of decavanadate anions and double-headed primary ammonium cation started to decompose from ca. 473 K (200 °C) [50]. In these cases, the organic ammonium cation was divalent and more strongly associated with the polyoxometalate anion than the monovalent C_12_NH_3_ cation. Therefore, we speculate that the C_12_NH_3_-CrMo_6_ hybrid crystal could start to decompose due to the removal of the C_12_NH_3_ cation at ca. 423 K (150 °C). The thermal stability of the C_12_NH_3_-CrMo_6_ hybrid crystal may be improved by blending C_12_NH_3_-CrMo_6_ with other polymers or glassy matrices to dilute the C_12_NH_3_-CrMo_6_ component. Although a more sophisticated fabrication process should be applied to improve the lasing efficiency, the C_12_NH_3_-CrMo_6_ hybrid crystal has shown potential as another category of inorganic–organic hybrid laser materials.

## 4. Materials and Methods

### 4.1. Materials

Chemical reagents including *n*-dodecylammonium chloride (C_12_NH_3_-Cl) were obtained from commercial sources (FUJIFILM Wako Pure Chemical Corporation, Osaka, Japan, and Tokyo Chemical Industry Co., Ltd. (TCI), Tokyo, Japan) and used without further purification. The sodium salt of CrMo_6_ (Na_3_[CrMo_6_O_18_(OH)_6_]·8H_2_O, Na-CrMo_6_) was prepared according to the literature [26,27]: Na_2_MoO_4_·2H_2_O (36.3 g, 150 mmol) was dissolved in 75 mL of H_2_O, and the solution pH was adjusted to 4.4 with HNO_3_. An aqueous solution (10 mL) containing 10 g of Cr(NO_3_)_3_·9H_2_O (25.0 mmol) was added to a pH-adjusted solution, and the solution was sequentially heated at 353 K for 1 h. The resultant supernatant was obtained by filtration and kept at room temperature for a week to isolate pink-purple block crystals of Na-CrMo_6_ (7.0 g, yield: 23% based on Mo).

### 4.2. Synthesis of C_12_NH_3_-CrMo_6_ Hybrid Crystal

An ethanol solution (30 mL) of C_12_NH_3_-Cl (0.11 g, 0.50 mmol) was added to an aqueous solution (30 mL) of Na-CrMo_6_ (0.34 g, 0.28 mmol) with heating at 333 or 343 K, and stirred for 5 min. The resultant suspension was filtrated and dried in the air to obtain a pale pink precipitate of C_12_NH_3_-CrMo_6_ (0.21–0.25 g, yield 45–54%). Single crystals of C_12_NH_3_-CrMo_6_ were grown from the synthetic filtrate kept at 315 or 323 K (0.01 g, yield ca. 2%). Some water molecules were removed from the crystal lattice in an ambient atmosphere. Anal. Calcd for C_36_H_94_N_3_CrMo_6_O_24_: C, 26.81; H, 5.87; N, 2.61%. Found: C, 26.85; H, 5.75; N, 2.69%. IR (KBr disk): 956 (m), 917 (s), 888 (s), 806 (w), 693 (m), 646 (s), 570 (w), 551 (w), 518 (w), and 416 (w) cm^−1^.

### 4.3. Measurements

Infrared (IR) spectra were measured using an FT/IR-4200ST spectrometer (Jasco Corporation, Tokyo, Japan, KBr pellet method). Powder X-ray diffraction (XRD) patterns were recorded using a MiniFlex300 diffractometer (Rigaku Corporation, Tokyo, Japan, Cu Kα radiation, *λ* = 1.54056 Å) under an ambient atmosphere. CHN (carbon, hydrogen, and nitrogen) elemental analyses were performed using a 2400II elemental analyzer (PerkinElmer, Inc., Waltham, MA, USA). Thermal gravimetric (TG) analyses were carried out on a TG/DTA-6200 (Seiko Instruments, Chiba, Japan) at a heating rate of 10 °C min^−1^ in a nitrogen atmosphere.

Steady-state diffuse reflectance, excitation, and emission spectra were recorded at 300 K using an FP-6500 fluorescence spectrometer (Jasco Corporation, Tokyo, Japan) equipped with an Xe lamp. Time-resolved emission spectra were obtained at 15 and 300 K with an Ultra CFR 400 YAG:Nd^3+^ laser (Big Sky Laser Technologies, Inc., Bozeman, MT, USA, 266 nm fourth harmonics, pulse duration 10 ns with a repetition rate of 10 Hz) as an excitation source. A Spectra Pro 2300i (Princeton Instruments, Inc., Trenton, NJ, USA) was utilized as a spectrometer, and a PI-Max with an intensified CCD camera (Princeton Instruments, Inc., Trenton, NJ, USA) was used as a detector. Pelletized samples of the C_12_NH_3_-CrMo_6_ precipitate were used for the aforementioned photoluminescence measurements.

### 4.4. X-ray Crystallography

Single crystal X-ray diffraction was measured with an XtaLAB PRO P200 diffractometer (Rigaku Corporation, Tokyo, Japan) using graphite monochromated Mo Kα radiation (λ = 0.71073 Å). The acquisition and processing of diffraction images including absorption correction were performed using CrysAlisPro (Version 1.171.39.46) [51]. Crystal structures were solved using SHELXT (Version 2018/2) [52] and refined by the full-matrix least-squares using SHELXL (Version 2018/3) [53]. The diffraction data recorded at the 2D beamline of the Pohang Accelerator Laboratory (PAL) confirmed the same crystal structure. CCDC 2312544.

## Figures and Tables

**Table 1:** Crystallographic data.

Compound	C_12_NH_3_-CrMo_6_
Chemical formula	C_36_H_98.63_N_3_CrMo_6_O_28.32_
Formula weight	1654.50
Crystal system	triclinic
Space group	*P*$\bar{1}$ (No. 2)
*a* (Å)	7.8562(4)
*b* (Å)	15.0256(6)
*c* (Å)	26.7754(13)
*α* (°)	94.034(4)
*β* (°)	91.871(4)
*γ* (°)	90.861(4)
*V* (Å^3^)	3150.7(3)
*Z*	2
*ρ*_calcd_ (g cm^−3^)	1.744
*T* (K)	103(2)
Wavelength (Å)	0.71073
*μ* (mm^−1^)	1.399
No. of reflections measured	53,171
No. of independent reflections	16,383
*R*_int_	0.1024
No. of parameters	724
*R*_1_ (*I* > 2*σ*(*I*))	0.0535
*wR*_2_ (all data)	0.1041

## Data Availability

Further details of the crystal structure investigation (CCDC 2312544) can be obtained free of charge via www.ccdc.cam.ac.uk/data_request/cif (accessed on 7 December 2023), or by emailing data_request@ccdc.cam.ac.uk, or by contacting The Cambridge Crystallographic Data Centre, 12 Union Road, Cambridge CB2 1EZ, UK; Fax: +44-1223-336033.

## Supplementary Materials

The supporting information can be downloaded at: https://www.mdpi.com/article/10.3390/ijms25010345/s1.
