# Supplementary material for: Photoluminescent Layered Crystal Consisting of Anderson-Type Polyoxometalate and Surfactant toward a Potential Inorganic–Organic Hybrid Laser
Source: Int J Mol Sci. 2023 Dec 26;25(1):345. doi: 10.3390/ijms25010345 (PMC10778674; doi:10.3390/ijms25010345)
Supplement: Supplementary file 1 [file ijms-25-00345-s001.zip › ijms-2789141-supplementary.pdf]

Supplementary material

Photoluminescent Layered Crystal Consisting of Anderson-Type Polyoxometalate and Surfactant toward a Potential Inorganic-Organic Hybrid Laser

Ayaka Mihara [1], Tatsuhiro Kojima [2], Yoriko Suda [3], Kyoka Maezawa [1], Toshiyuki Sumi [1], Naoyuki Mizoe [1], Ami Watanabe [1], Hironori Iwamatsu [4], Yoshiki Oda [5], Yosuke Okamura [4], and Takeru Ito [1,*]

[1] Department of Chemistry, School of Science, Tokai University, Hiratsuka, Kanagawa 259-1292, Japan

[2] Department of Applied Chemistry, Kobe City College of Technology, Kobe, Hyogo 651-2194, Japan

[3] Department of Electric and Electronic Engineering, School of Engineering, Tokyo University of Technology, Hachioji, Tokyo 192-0982, Japan

[4] Department of Applied Chemistry, School of Engineering, Tokai University, Hiratsuka, Kanagawa 259-1292, Japan

[5] Technology Joint Management Office, Tokai University, Tokai University, Hiratsuka, Kanagawa 259-1292, Japan

* Corresponding author. E-mail address: takeito@tokai.ac.jp

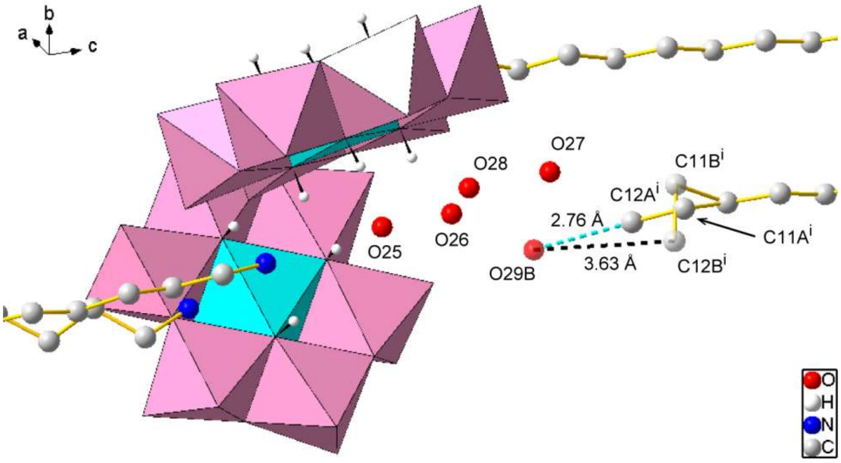

Figure S1. Molecular conformation around the disordered part (C: gray, N: blue, O: red, H: white). $CrMo_6$ anions are depicted in polyhedral model (Mo: pink, Cr: light blue). H atoms of $C_{12}NH_3$ cations and solvents are omitted for clarity. The disordered atoms (C11B, C12B, and O29B) in the minor part are indicated in transparent color. Black broken line indicates a short contact due to C–H···O hydrogen bonding. Light blue broken line represents a short distance (not a short contact, see text in detail). Symmetry code: (*i*) *x*, *y*, 1+*z*.

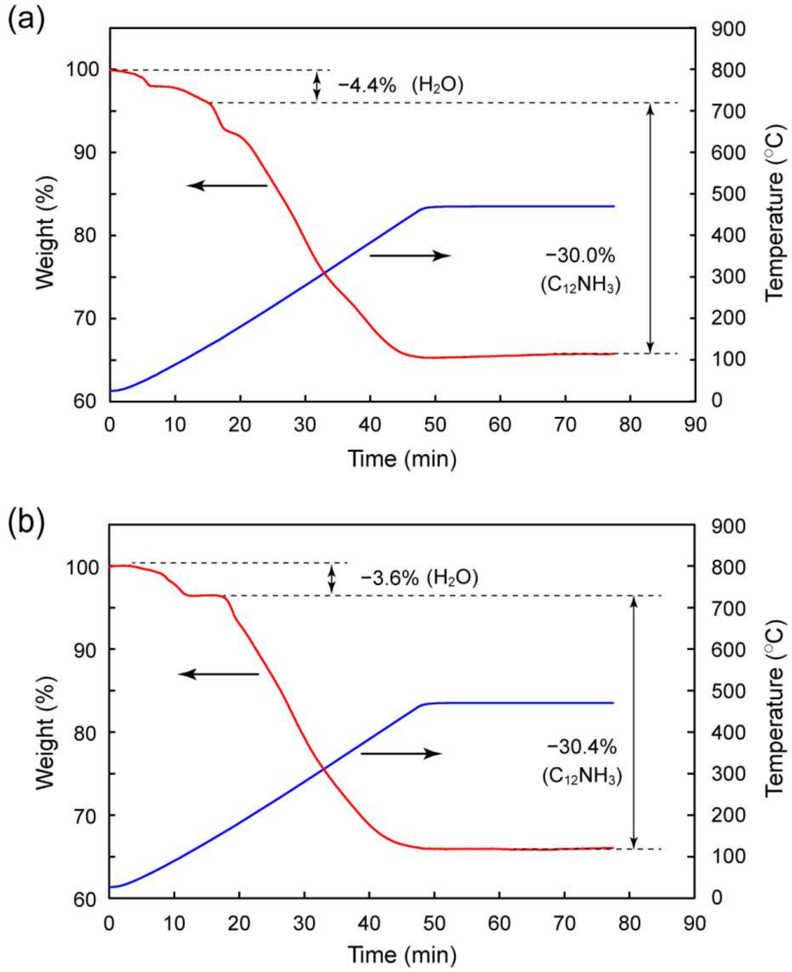

Figure S2. TG profiles of $C_{12}NH_3$-$CrMo_6$ hybrid crystal: **(a)** Precipitate of $C_{12}NH_3$-$CrMo_6$; **(b)** Single crystal of $C_{12}NH_3$-$CrMo_6$. Conditions: rt – 500 °C (rate: 10 °C/min, 30 min hold at 500 °C), N_2 flow (100 mL/min).
